# Edoxaban in patients with non-valvular atrial fibrillation after percutaneous coronary intervention: ENCOURAGE-AF design

**DOI:** 10.1038/s41598-023-44345-7

**Published:** 2023-10-25

**Authors:** Stephan Baldus, Jan Beyer-Westendorf, Helge Möllmann, Wolfgang Rottbauer, Elisabeth Beyerlein, Andreas Goette

**Affiliations:** 1 Heart Center of the University Hospital Cologne, University Hospital Cologne, Universitätsklinikum Köln, Kerpener Str. 62, 50937 Cologne, Germany; 2 Universitätsklinikum Carl Gustav Carus Dresden, Dresden, Germany; 3 St. Johannes Hospital Dortmund, Dortmund, Germany; 4 Universitätsklinikum Ulm, Ulm, Germany; 5 Daiichi Sankyo Europe GmbH, Munich, Germany; 6 St. Vincenz Krankenhaus Paderborn, Paderborn, Germany

**Keywords:** Environmental impact, Environmental impact

## Abstract

Approximately one fifth of patients diagnosed with atrial fibrillation (AF) undergo a percutaneous coronary intervention (PCI). Current guidelines recommend different combinations and durations of triple or dual antithrombotic therapy for these patients but data on the implementation of these recommendations in clinical routine are scarce. ENCOURAGE-AF is a prospective, non-interventional, non-comparative, multicentre study. Approximately 720 patients will be consecutively enrolled from 70 participating sites across Germany. Patients with non-valvular AF treated with edoxaban, who have undergone successful PCI, have no planned elective cardiac intervention during the study period, have capability, availability, and willingness for follow-up by telephone interview during the study, are aged ≥ 18 years with life expectancy ≥ 1 year, and provide written informed consent, will be included. Eligible patients will be enrolled between 4- and 72-h after completing a successful PCI. Duration of exposure to and dosing regimens of edoxaban, antiplatelet agents and other concomitant medications of interest will be monitored in line with the clinical practice. Physician- and patient-reported clinical events, adverse drug reactions, patient quality of life (EQ-5D-5L) and health resource utilisation (HRU) parameters will be evaluated at 30 days and 1-year post-PCI. The ENCOURAGE-AF non-interventional study will provide insights into the patterns of edoxaban usage in combination with antiplatelet treatment and other concomitant medications in AF patients with a successful PCI over a 1-year time period during routine clinical practice in Germany. The effectiveness and safety of edoxaban in this patient population, as well as patients’ quality of life and HRU will be evaluated.

**Trial registration**: Clinicaltrial.gov NCT04519944, registered on 20 August 2020.

## Introduction

Atrial fibrillation (AF), the most prevalent cardiac arrhythmia, is estimated to affect 17.9 million Europeans by 2060^1^. Approximately 20% of patients with AF will require a percutaneous coronary intervention (PCI) for the treatment of obstructive coronary artery disease (CAD)^2^. To avoid post-procedural cardio-embolic and coronary events, it is recommended that patients with AF who undergo PCI receive a combination of both oral anticoagulation (OAC) and antiplatelet therapy^3^.

In the last two decades, non-vitamin K antagonist oral anticoagulants (NOACs) have been introduced as alternatives to the standard vitamin K antagonist (VKA) therapy for patients with AF. Four large randomised controlled trials (RCTs) investigating the efficacy and safety of dual antithrombotic therapy (NOAC + P2Y_12_ inhibitor) versus triple therapy (OAC [VKA or NOAC] + P2Y_12_ inhibitor + aspirin) have been conducted with AF patients undergoing PCI so far^4–8^. These trials collectively demonstrated lower rates of clinically significant bleeding with NOAC-based dual therapy versus the standard VKA-based triple therapy^9–12^.

Aspirin use as part of a triple therapy regimen has been implicated in the prevention of stent thrombosis and myocardial infarction (MI) events in patients with acute coronary syndrome (ACS) undergoing PCI with an elevated risk of recurrent ischaemic events^13^. A sub-analysis from the AUGUSTUS trial with apixaban demonstrated that the trade-off between an increase in severe bleeding events and reduction in severe ischaemic events can be balanced if aspirin is prescribed immediately after and up to 30 days post-PCI^14^.

Based on the available evidence, the current European Society of Cardiology 2020 guidelines for AF^3^ recommend that AF patients with ACS or chronic coronary syndrome (CCS) receive NOAC-based triple therapy (clopidogrel is preferred with ACS) for a maximum of 1 week after an uncomplicated PCI, in patients with low risk of stent thrombosis, or a bleeding risk that outweighs the risk of stent thrombosis, irrespective of the type of stent used. This can be extended (NOAC plus aspirin plus clopidogrel) for ≤ 1 month if the risk of stent thrombosis exceeds the bleeding risk. Continued administration of dual therapy is advised up to 12 months in patients with ACS (NOAC plus clopidogrel preferred) or 6 months with CCS (NOAC plus clopidogrel) after an uncomplicated PCI. In any case, the individual risk assessment and the treatment protocol should be clearly specified at hospital discharge.

Edoxaban has gained authorisation in the European Union for the treatment of adult patients with AF for the prevention of ischaemic stroke and systemic embolism (SE)^15^. Indeed, the safety of edoxaban-based dual antithrombotic therapy was recently evaluated in comparison with VKA-based triple antithrombotic therapy in patients with AF who underwent PCI during the ENTRUST-AF PCI trial^7^. The edoxaban-based regimen was non-inferior to the VKA-based regimen with respect to bleeding events, and no significant differences in the incidences of ischaemic events were observed^7^. However, at present, data describing the use, effectiveness and safety of dual antithrombotic therapy with edoxaban in patients with AF undergoing PCI in a real-world setting are limited.

The ENCOURAGE-AF non-interventional study will aim to complement RCT findings by facilitating a better understanding of the patterns of edoxaban use in combination with antiplatelet therapies in AF patients undergoing PCI during routine clinical practice in Germany. The effectiveness and safety of edoxaban use in this setting will also be evaluated up to one-year post-successful PCI. The present article will summarise the rationale and design of the ENCOURAGE-AF study.

## Methods

### Study design

#### Study objectives and outcome measures

The primary objective of ENCOURAGE-AF is to gain insights into the peri- and post-procedural use of anticoagulation and antiplatelet therapy in combination with edoxaban in patients with non-valvular atrial fibrillation (NVAF) undergoing successful PCI in German routine clinical practice for up to 1 year after the procedure. The duration of exposure to and dosing regimens of the antithrombotic agents and other concomitant medications of interest used will be evaluated and any patterns of treatment use will be reported.

The secondary objective is to investigate the effectiveness and safety of edoxaban in combination with antiplatelet therapy up to 1 year after a successful PCI by reporting incidences of physician- and patient-reported clinical events of interest (bleeding events, stroke, MI, PCI, or death) and documented adverse drug reactions (ADRs). Additional secondary objectives include evaluating patients’ health-related quality of life via the five-level EQ-5D (EQ-5D-5L) questionnaire and gaining insights into patients’ health resource utilisation (HRU) with respect to hospital admissions and number of days of hospitalisation.

#### Participating sites

Study sites were identified using the Daiichi Sankyo Deutschland GmbH internal database. During the feasibility process, 120 sites were contacted with an aim of selecting 60 participating sites. Each site was asked to complete a detailed feasibility questionnaire and participate in a short phone call to discuss the feasibility of the study. All sites could freely choose to accept or refuse participation in the study. Site selection was based on a defined set of criteria (Table 1). A stepwise process was employed to ensure a representative regional distribution of sites and site specialties were chosen.

**Table 1:** Site selection criteria.

Site selection criteria
Having access to AF patients undergoing PCI (≥ 20 patients in the planned recruitment time)
Having patients treated with edoxaban
Agreeing to follow-up each patient for approximately a 1-year period according to a clinical routine, performing structured phone interviews ca. 1-month and 1-year after successful PCI
Being able to document data in English language
Being able to complete the study using the electronic data capturing system
Being able to conduct the study adequately, with enough time and staff to identify eligible patients, conduct the patient consent process, participate in required trainings, and follow-up with study-related activities

*PCI* percutaneous coronary intervention.

Patient enrolment commenced in July 2020. Selected sites are undertaking consecutive patient enrolment and are estimated to contribute 20 patients each. To assess the representativeness of the study population, each site is required to complete a screening log for eligible patients, documenting the reasons for patient inclusion or exclusion.

#### Study population and eligibility

At study start, the study was planned to include 1200 patients enrolled from approximately 60 sites with PCI capability until end of 2021 across Germany. Due to the Coronavirus-19 pandemic, the patient recruitment was considerably slower than expected. Therefore, it was decided to prolong the recruitment for six months until end of June 2022 and decrease the sample size to 720 patients. Patients who fulfil the eligibility criteria listed in Table 2 qualify for study enrolment. For those with a staged PCI planned, eligibility will be assessed after completion of the last successful PCI.

**Table 2:** Patient eligibility criteria.

Patient eligibility criteria
Providing written informed consent for participation in the study
Having NVAF treated with edoxaban
Undergone successful PCI
Having no planned elective cardiac intervention for the entire study duration (up to 1 year)
Capability, availability, and willingness for follow-up by telephone by the site for the entire study duration (up to 1 year)
No simultaneous participation in any other interventional study
≥ 18 years of age
Life expectancy > 1 year

*NVAF* non-valvular atrial fibrillation, *PCI* percutaneous coronary intervention.

Given that ENCOURAGE-AF is a non-interventional study, there are no specific study exclusion criteria. To allow for a meaningful differentiation between elective PCI and ACS populations, recruitment is monitored and, if necessary, adjusted in some sites to ensure that at least 25% of all enrolled patients underwent PCI for ACS.

#### Schedule

**Figure 1:**
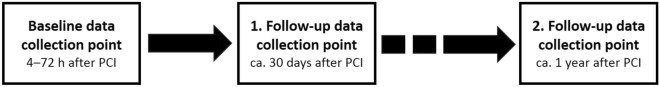
Study flow chart. *PCI* percutaneous coronary intervention.

A flow chart detailing the pre-specified data collection time points is shown in Fig. 1 and the schedule for data collection, which will be recorded in the electronic Case Report Form (eCRF), is presented in Table 3. The observational period for each patient will begin between 4- and 72-h post-removal of the guiding catheter of a successful PCI. Patient-specific demographics and characteristics will be recorded at the time of enrolment (referred to as baseline herein; Table 3); the full list of the recorded variables is available in Supplemental Appendix SI. Patients will also be required to complete an EQ-5D-5L questionnaire at baseline to evaluate quality of life parameters.

**Table 3:** Data collection schedule.

	Baseline	30-day Follow-up	1-year Follow-up
Date	✓	✓	✓
Eligibility	✓		
Demographics	✓		
Vital signs	✓		
Relevant medical history	✓		
Relevant concomitant diseases	✓		
Coronavirus disease-2019 status	✓	✓	✓
Relevant concomitant medication^1^	✓	✓	✓
Intake of edoxaban	✓	✓	✓
Risk factors (CHA_2_DS_2_-VASc, HAS-BLED Score)	✓		
Current status of NVAF	✓		
Creatinine, labile INR^2^	✓		
PCI details^3^	✓		
HRU parameters^4^	✓	✓	✓
PRO (EQ-5D-5L)	✓		✓
Clinical events of interest^5^	✓	✓	✓
Suspected edoxaban ADRs	✓	✓	✓

*ADR* adverse drug reaction, *ASA* acetylsalicylic acid, *HRU* health resource utilisation, *MI* myocardial infarction, *NOAC* non-vitamin K antagonist oral anticoagulant, *NVAF* non-valvular atrial fibrillation, *PCI* percutaneous coronary intervention, *PRO* patient reported outcome, *VKA* vitamin K antagonist.

^1^Relevant concomitant medication: heparin, P2Y_12_ inhibitor, ASA, NOAC, GP IIb/IIIa inhibitor, VKA, antiarrhythmic/rate control medication, NSAIDs, proton pump inhibitor, hormone therapy, P-gp inhibitor and insulin.

^2^Last available measurement in medical records (baseline or earlier).

^3^PCI Details: indication for PCI, access for PCI, number of drug-eluting stents, length, diameter and location of each stent, start date, stop date and time of procedure.

^4^HRU parameters: hospital admissions and length of hospital stay.

^5^Bleeding events, stroke, MI, PCI and death. Patients may document the events in the patient memory aid, if available. The recorded parameters, listed fully in Supplemental Appendix SI, include clinical events of interest, relevant concomitant medications, ADRs and HRU parameters.

Each patient will be followed-up at approximately 30-days (± 7 days) and 1-year (± 2 weeks) post-PCI (Table 3). Patients will be contacted by their physician by telephone to collect all relevant available information relating to the patients’ routine management between the respective data collection points. If a patient is not available, physicians are permitted to contact relatives or their primary care doctor. The recorded parameters, listed fully in Supplemental Appendix SI, include clinical events of interest, relevant concomitant medications, ADRs and HRU parameters. The quality-of-life assessment questionnaire, EQ-5D-5L, will also be collected at the 1-year follow-up.

To facilitate complete and accurate data reporting, a structured phone interview guide will be distributed to the treating physicians prior to conducting the follow-up appointments. During the observation period, patients will have the option to complete a hardcopy memory aid diary to assist with recalling information relevant to the study at follow-up. Further, to allow for the alternative of an electronic diary, a study app for patients was developed in 2021. The patient can use the study app to document changes of the most important medications (edoxaban, clopidogrel and ASS) during the study and report clinical events of interest including hospitalisations during the 1-year follow-up period. Each patient entry can be sent to the respective study site via an email assuring that the site is immediately informed to enable immediate patient contact or to guide the structured phone interview at 1-year. The handling of data provided by the app is at the discretion of the site physician. In addition, the patient can use the app to complete the EQ-5D-5L at 1-year follow-up. These data will be automatically sent to the study database.

In the case that a patient cannot be reached at the pre-specified data collection time points (ca. 30-days and/or ca. 1-year), the treating physician is expected to repeatedly attempt to contact the patient in close proximity to the missed time point. If a patient cannot be contacted at the ca. 30-day time point, the physician is advised to continue to persistently attempt to reach the patient at the 1-year time point.

Discontinuation of edoxaban for any reason during the follow-up period will be recorded in the eCRF and at 1-year follow-up documentation by the treating physician. If edoxaban was discontinued due to an ADR, the patient must be followed-up until the ADR is resolved. Details relating to any subsequent treatment with other-than-baseline anticoagulants will be documented (i.e., type, primary cause). In the case that a patient terminates the study early, the treating physician is requested to document the primary cause for early study termination.

In accordance with the Declaration of Helsinki and other applicable regulations, all patients have the right to withdraw consent from participation in the study at any time and for any reason without prejudice to his or her future medical care by the physician or at the institution. In this case, the treating physician will complete and report the observations as thoroughly as possible up to the date of consent withdrawal and including the date of the last dose of relevant concomitant medication and edoxaban intake. The reason for consent withdrawal (if given) will be documented in the eCRF.

#### Data collection

All data collected at baseline and during the follow-up will be entered into the eCRF by designated, trained personnel on-site. Data collected from patients at enrolment, from medical records maintained during routine clinical practice and from telephone interviews will be included. No visits, examinations, laboratory tests or procedures are mandated as part of the study. Participating sites are expected to submit patient’s data within 5 working days of each specified time point (baseline, ca. 30 days and ca. 1 year after enrolment).

Appropriate training on the use of the online data capture system and eCRF completion is provided to each participating site. Each data item captured in the eCRF is reviewed at the site and electronically signed-off by the treating physician. Any changes or corrections to eCRFs are documented in an audit trail for which an adequate explanation is required.

#### Data management

Metronomia Clinical Research GmbH (Munich, Germany) are responsible for the setup and maintenance of the eCRF, the creation and execution of the data management plan and for providing biostatistics support. All data will be collected and managed using the Electronic Data Capture (EDC) software, Clincase version 2.7 (Quadratek Data Solutions Ltd., Berlin, Germany). eCRF data entered into Clincase will be transferred into an Oracle™ version 9.2 (Oracle Corporation, Austin, TX, USA) database to facilitate medical coding. For concomitant medications, coding will be performed using the actual World Health Organization Drug Dictionary. ADRs will be coded in the safety database using the latest version of Medical Dictionary for Regulatory Activities. A coding review will be conducted prior to each database snapshot or database lock for the final analysis, respectively.

Data validation and cleaning will take place on a regular, ongoing basis (at least twice weekly). Queries flagged by edit checks or a manual reviewer (a Metronomia data manager or Clinical Research Associate [CRA]) identifying missing, invalid, or inconsistent data will be forwarded to the relevant site for resolution. Query responses will be reviewed to ensure that they are addressed appropriately. Quality control checks will be performed by Metronomia on 3% of all manually closed queries. CRAs will perform source document verification (SDV) of the data.

eCRFs will be entry locked on an individual basis once the following conditions are met: (i) study is completed by subject, (ii) SDV is completed and (iii) all data queries are resolved. Data cleaning will increase prior to obtaining database snapshots for the baseline and 30-day interim analyses and the final data lock at 1-year with the aim of achieving a minimum of 90% data cleanliness, depending on the use of the data. SAS version 9.4 (SAS Institute Inc., Cary, NC, USA) or higher will be used to complete the analyses. For direct transcription of Clincase data into SAS data files, the ‘Clincase SAS Transfer’ tool will be used.

The study app, developed by 3m5 Media GmbH, Dresden, runs on all popular iOS and android smartphones. It uses an interface with the eCRF to allow electronic onboarding and data exchange with the patient-specific eCRF pages. The study site enters a site identifier (ID), patient ID and a personal identification number provided by the eCRF into the app, resulting in the direct connection of the app with both the eCRF and the site email address.

#### Definitions

##### Concomitant medications

Concomitant medications considered relevant to the study at baseline and the follow-up time points are listed in Table 4.

**Table 4:** Relevant concomitant medications documented at baseline and each follow-up time point.

Concomitant medication	Baseline	Follow-up^1^
Heparin	✓	
Aspirin	✓	✓
P2Y_12_-inhibitors	✓	✓
NOACs	✓	✓ Edoxaban only
GP IIb/IIIa inhibitors	✓	
VKAs	✓	
Antiarrhythmics/rate control drugs	✓	✓
NSAIDs	✓	✓
P-gp inhibitors	✓	✓
Proton pump inhibitors	✓	✓
Hormone therapy	✓	✓
Insulin	✓	

The following information was documented for each medication: daily dose, unit, start date, stop date or ongoing. For insulin, whether a patient did or did not take the medication was noted (yes, no or unknown).

*NOAC* non-vitamin K antagonist oral anticoagulant, *PCI* percutaneous coronary intervention, *VKA* vitamin K antagonist.

^1^Patients were followed-up after ca. 30 days or ca. 1 Year.

##### Bleeding events

Since bleeding event data will be collected in a different setting at baseline compared with the follow-up period, different methods for categorising bleeding events will be employed. At baseline, bleeding events will be recorded in a hospital setting and will be classified according to the International Society on Thrombosis and Haemostasis (ISTH) bleeding criteria (major, clinically relevant non-major [CRNM] or minor events^16,17^. At follow-up, patients will be questioned regarding bleeding events via telephone, therefore a different set of patient friendly categories will be used (Table 5). The patient memory aid will describe the different bleeding categories for ease of understanding. A patient’s relatives or primary care physician may be contacted to confirm death due to bleeding.

**Table 5:** Bleeding event categories for outcome.

Evaluation	Bleeding event category
Relative or primary care doctor reported death due to bleeding	Bleeding with fatal outcome
Questions regarding hospitalisation and blood transfusions for bleeding are answered with “yes”	Bleeding requiring hospitalisation and blood transfusion
Question regarding hospitalisation for bleeding is answered with “yes”	Bleeding requiring hospitalisation
Question regarding outpatient medical intervention is answered with “yes”	Bleeding requiring medical intervention
All questions regarding bleedings are answered with “no”	Bleeding not requiring medical intervention

##### Stroke

A clinical event characterised by an acute onset of a focal neurological deficit, usually in the distribution of a single brain artery (including the retinal artery), which is not attributable to an identifiable non-vascular cause. The deficit must be associated with symptoms lasting more than 24 h or result in death within 24 h of symptom onset^18^. The following stroke categories will be recorded: any stroke; ischaemic stroke; haemorrhagic stroke; stroke (unknown type).

##### MI

Where possible, spontaneous and periprocedural MI events are defined in accordance with the previously reported Universal and Society for Cardiovascular Angiography and Interventions definitions respectively^19,20^.

##### Successful PCI

A successful PCI procedure is characterised by the following angiographic features:

(1) a minimum stenosis diameter of < 20% (residual blockage or stenosis reduced to < 20% of the artery’s diameter) and (2) sufficient enlargement of the lumen at the target site to improve coronary artery blood flow with final Thrombolysis in Myocardial Infarction flow grade 3, without occlusion of a significant side branch, flow-limiting dissection, distal embolisation, or angiographic thrombus.

##### Death

Deaths will be categorised as cardiovascular (CV) or all-cause death, with the aim of capturing the primary cause of death. Where possible, CV death will be classified in accordance with the criteria specified by the Academic Research Consortium^21^. The primary cause of death may be distinct from both the mode of death and an intervening cause that is temporally closer and contributes to the death.

##### Patient reported outcome (PRO): EQ-5D-5L questionnaire

EQ-5D-5L, developed by the EuroQoL group^22^, is a generic utility score ranging from 0 (very poor) to 1 (excellent) that rates current, overall health states () based on five domains: mobility, self-care, usual activities, pain or discomfort, and anxiety or depression. Patients also rate their current state of health on a vertical visual analogue scale of 0 (very poor) to 100 (excellent).

#### Quality control

This study will be conducted according to the rules of ‘Good Pharmacoepidemiology Practice’ and the ‘Guidelines on Good Pharmacovigilance Practices’. The clinical research organisation SSS International Clinical Research GmbH (Munich, Germany) will be responsible for managing and performing all operational tasks, including setting-up, monitoring and managing the non-interventional study.

Regular data quality checks will be performed throughout the study to ensure the accuracy and completeness of recorded data, the satisfactory protection of patients’ rights and compliance with the observational plan and relevant regulatory requirements. The data management team and the medical monitor will direct any queries relating to the eCRFs to the relevant centres.

The integrity of all submitted data will be verified by signature by the responsible physician.

On-site monitoring will be performed at 20% of randomly selected sites. During the on-site visits, all patient informed consent documentation will be verified and the submitted data for 3 randomly selected patients will be quality checked against their medical records.

#### Sample size calculations

As ENCOURAGE-AF is a non-interventional study that will collect routine clinical data only, no primary parameter was defined for the sample size calculation. A sample size of 720 patients will provide sufficient precision (measured by width of 95% confidence interval [CI]) for the rates of major and CRNM Bleeding, CV death, stroke (any, ischemic, haemorrhagic) and MI during the one-year follow-up. In the randomised controlled clinical study (ENTRUST-AF PCI), an event rate of 17% was observed for the composite outcome of major or CRNM bleeding (ISTH) in patients receiving edoxaban^7^. With approximately 720 enrolled patients it will be possible to estimate the 95% CI with a precision range of ± 2.7% (resulting in relative precision of 16.1%).

#### Statistical analysis

The patient disposition will include patients from the *All-Documented Patient Set (APS),* who have a signed and dated an informed consent form. This will summarise the number and percentage of patients in the different analysis sets including reasons for exclusion, who completed or did not complete the regular observational period (including reasons for non-completion), as well as patients who did or did not receive at least one dose of edoxaban post Index PCI. The *Baseline Analysis set* (BAS; all patients from the APS who fulfil the study eligibility criteria with any further baseline documentation) will be used to describe patient demographics and other baseline characteristics shown in Table 3 (and listed in full in Supplemental Appendix SI).

All other statistical analyses will be performed on data from the *Full Analysis Set (FAS),* consisting of all patients from the *BAS* with documentation from at least one time point post-baseline. SAS version 9.4 or higher will be used to conduct the analyses.

To address the primary objective, a descriptive analysis of data on the usage of peri- and post-procedural antithrombotic therapies, as well as other relevant concomitant medications at baseline and the two follow-up time points will be performed (to include extent of exposure and dosing regimens).

The analysis for the secondary objectives will be based on data documented for physician- and patient-reported clinical events of interest (bleeding events, stroke, MI, PCI, or death), ADRs, PROs (EQ-5D-5L questionnaire) and HRU parameters. Data for clinical events of interest will be reported as absolute and relative frequencies with 95% confidence intervals and Kaplan–Meier analyses will be performed where applicable to determine outcome risk over time. ADRs will be coded using the Medical Dictionary for Regulatory Activities coding dictionary (version 23.1 or higher) and ADR incidences will be reported. EQ-5D-5L and HRU parameters will be summarised descriptively.

### Ethics approval and consent to participate

This non-interventional study fulfils the requirements of the Directive 2001/83 EC, Guidelines on Good Pharmacovigilance Practices, Directive 95/46 EC and the Declaration of Helsinki and will be conducted in accordance with the respective standard operating procedures of Daiichi Sankyo Europe GmbH (DSE) and/or Clinical Research Organisation in charge of the non-interventional study. The study was approved by the Leading Ethics Committee of University of Cologne on 17th July 2020. The Notification to Bundesinstitut für Arzneimittel und Medizinprodukte was confirmed on 21st July 2020. A site-specific Amendment (Observational Plan and Informed Consent Form) was approved for the study app by the Ethics Committee of Sachsen on 1st June 2021. Written informed consent will be obtained from all patients. If the patient uses the study app, a separate written informed consent for the study app will be obtained.

## Discussion

An antithrombotic therapeutic strategy consisting of an OAC and dual antiplatelet therapy (DAPT; P2Y_12_ inhibitor ± aspirin) is recommended in AF patients undergoing PCI^3^. DAPT is administered with the aim of reducing the risk of post-procedural stent thrombosis during the healing process, as well as other atherothrombotic events; however, the prolonged use of DAPT incurs an increased bleeding risk^23^. Results from the ENCOURAGE-AF study will help understand the current use of antithrombotic therapies (as well as other concomitant medications) in AF patients undergoing PCI during routine clinical practice in Germany. Given the uncertainties around the optimal use and duration of aspirin use following a PCI procedure in AF patients, ENCOURAGE-AF will provide valuable real-world data on the combination and duration of NOACs and antiplatelet agents in patients with AF undergoing PCI. Results from both the AUGUSTUS and ENTRUST AF-PCI trials suggest that concomitant aspirin use is necessary post-PCI, although the optimal duration of concomitant use is not clear yet^13,14^. Investigating the patterns of use of antithrombotics during the first 30-days after the procedure is of particular interest since it is during this time when the risk/benefit trade-off between dual and triple therapy is most important^14^. This is reflected in the most recent ESC 2020 guidelines for patients with AF^3^.

NOAC-based dual therapy is associated with a long-term reduction in bleeding events over the course of the first year of treatment compared with VKA-based triple therapy, however, the short-term use of triple therapy is recommended immediately after a successful PCI to reduce the risk of stent thrombosis^3^. Depending on a patient’s risk of stent thrombosis versus risk of bleeding, the recommended duration for aspirin therapy in parallel with NOAC plus clopidogrel ranges from a maximum of 1 week (bleeding risk > stent thrombosis risk) up to a maximum of 1 month (stent thrombosis risk > bleeding risk)^3^. The ongoing RIVA-PCI study (ClinicalTrial.gov identifier: NCT03315650) will also investigate the real-world use of antithrombotics in AF patients with ACS (or stable disease) undergoing PCI. Adherence to antithrombotic treatment and any complications associated with treatment will be evaluated over 14-months in patients receiving rivaroxaban. Together with the RIVA-PCI registry, ENCOURAGE-AF will expand our knowledge of the patterns of use of antithrombotic therapy in German AF patients with PCI in routine clinical practice. Data from the two non-interventional studies could highlight areas where additional medical education is needed to ensure patients with AF and stent implantation receive optimal clinical care in Germany.

ENCOURAGE-AF will complement the findings of the ENTRUST-AF PCI trial by providing valuable information on the real-world effectiveness and safety of edoxaban in an expanded pool of patients, many of whom may not have otherwise been eligible for entry into the RCT. The study will also provide insights into the quality of life of AF patients following PCI which has not been widely investigated. Moreover, data on the HRU of patients in this specialised patient population will be explored. Insights into the quality of life and health resource utilisation of patients in this specialised population will also be presented and discussed.

The ENCOURAGE-AF study is strengthened by the absence of exclusion criteria, which will avoid selection bias and facilitate the habitual documentation of routine clinical practice. Thus, the prescribing behaviour of physicians will not be influenced by the study. As with all real-world studies, the ENCOURAGE-AF study design has several limitations. Patients participating in competing interventional trials or other edoxaban non-interventional studies are not eligible for inclusion in the study, which might inadvertently lead to some degree of selection bias. Furthermore, the analyses evaluating the effectiveness and safety of edoxaban, as well as effects on patients’ quality of life and HRU, will not include a control group for comparisons, which could lead to bias in the interpretation of the outcomes.

As with all observational studies, ENCOURAGE-AF faces the risk of under-reporting of data, especially due to the long time period between the 1-month and 1-year follow-up time points. This can potentially result in the misclassification of treatment exposure and the incorrect documentation of treatment changes, as well as all other outcomes. While patient medical records, the optional hardcopy patient memory aid, and provision of the structured telephone interview guide for physicians will support the optimal documentation of data between the follow-up time points, their influence on under-reporting relies upon the accuracy and precision of the data recorded. The implementation of a study app that is easy to install and use on older iOS and android smartphones, and which provides immediate email feedback to the study site, is expected to reduce the risk of under-reporting and misclassification. Furthermore, the direct transfer of EQ-5D-5L data from smartphone to the eCRF will reduce the risk of missing data and site data entry errors.

Throughout the study, different methods for categorising bleeding events will be used at baseline compared with the follow-up period due to the different settings in which the data will be collected (hospital versus telephone respectively). During the follow-up, a number of factors may influence the accuracy of the reporting, which relies upon both the physicians’ interviewing techniques and the ability of patients to recall past events precisely, via telephone. The accuracy of reporting could be affected by a patient’s age and/or health status. Thus, in contrast to baseline, when bleeding events are reported in hospital, bleeding events may be under-reported during the follow-up.

## Conclusions

The ENCOURAGE-AF non-interventional study will provide insights into the use of antithrombotics in patients with AF undergoing successful PCI over a year of routine care in Germany. Valuable information regarding the patterns of edoxaban in combination with antiplatelet treatment and other concomitant medications will be obtained for this patient population. Furthermore, the effectiveness and safety of edoxaban, as well as patients’ quality of life and HRU will be evaluated.

### Supplementary Information

Supplementary Information 1.

## Data Availability

All data generated or analysed during this study are included in this published article (and its Supplementary Information file).

## Abbreviations

ACS: Acute coronary syndrome
ADR: Adverse drug reaction
AF: Atrial fibrillation
APS: All documented patient set
BAS: Baseline analysis set
CAD: Coronary artery disease
CCS: Chronic coronary syndrome
CRNM: Clinically relevant non-major
CV: Cardiovascular
DAPT: Dual antiplatelet therapy
eCRF: Electronic case report form
FAS: Full analysis set
HRU: Health resource utilisation
ID: Identifier
MI: Myocardial infarction
NOAC: Non-vitamin K antagonist oral anticoagulant
OAC: Oral anticoagulant
PCI: Percutaneous coronary intervention
PRO: Patient-reported outcome
RCT: Randomised clinical trial
VKA: Vitamin K antagonist
