# Supplementary material for: Edoxaban in patients with non-valvular atrial fibrillation after percutaneous coronary intervention: ENCOURAGE-AF design
Source: Sci Rep. 2023 Oct 25;13:18215. doi: 10.1038/s41598-023-44345-7 (PMC10600182; doi:10.1038/s41598-023-44345-7)
Supplement: Supplementary file 1 — Supplementary Information 1. [file 41598_2023_44345_MOESM1_ESM.docx]

# Supplemental Appendices

## Supplemental Appendix I: Study variables and data collection schedule

|  | **Baseline** | **30 Day**  **Follow-up** | **1 Year**  **Follow-up** |
| --- | --- | --- | --- |
| **Date** | ✓ | ✓ | ✓ |
| **Informed Consent** | ✓ |  |  |
| **Eligibility** | ✓ |  |  |
| **Demographic variables** | ✓ |  |  |
| **Vital signs** | ✓ |  |  |
| **Creatinine and labile INR^1^** | ✓ |  |  |
| **Relevant medical history** |  |  |  |
| ACS | ✓ |  |  |
| Unstable angina | ✓ |  |  |
| Acute NSTEMI | ✓ |  |  |
| Acute STEMI | ✓ |  |  |
| Bypass surgery (number) | ✓ |  |  |
| PCI (number) | ✓ |  |  |
| Stroke | ✓ |  |  |
| TIA | ✓ |  |  |
| Systemic embolism | ✓ |  |  |
| VTE | ✓ |  |  |
| Bleeding tendency or predisposition | ✓ |  |  |
| ISTH major, CRNM or minor bleeding before PCI | ✓ |  |  |
| **COVID-19 status** | ✓ | ✓ | ✓ |
| **Relevant concomitant diseases** |  |  |  |
| Diabetes | ✓ |  |  |
| Hypertension | ✓ |  |  |
| Excess alcohol use | ✓ |  |  |
| COPD | ✓ |  |  |
| Congestive HF | ✓ |  |  |
| Dyslipidemia | ✓ |  |  |
| Peripheral artery disease | ✓ |  |  |
| Abnormal liver function | ✓ |  |  |
| Abnormal renal function | ✓ |  |  |
| Congenital or acquired disease  related to bleeding | ✓ |  |  |
| VHD | ✓ |  |  |
| **Risk factors** |  |  |  |
| CHA_2_DS_2_-VASc | ✓ |  |  |
| HAS-BLED Score | ✓ |  |  |
| **Current status of NVAF** |  |  |  |
| Paroxysmal AF | ✓ |  |  |
| Persistent AF | ✓ |  |  |
| Long-standing persistent AF | ✓ |  |  |
| Permanent AF | ✓ |  |  |
| **PCI Details** |  |  |  |
| Indication for PCI | ✓ |  |  |
| Access for PCI | ✓ |  |  |
| Number, diameter, length, and location of drug-eluting stents | ✓ |  |  |
| Start date and time and stop date and time | ✓ |  |  |
| **Relevant concomitant medication^2^** |  |  |  |
| Heparin | ✓ |  |  |
| Aspirin | ✓ | ✓ | ✓ |
| P2Y_12_-inhibitors | ✓ | ✓ | ✓ |
| NOACs | ✓ | Edoxaban only | Edoxaban only |
| VKA | ✓ |  |  |
| GP IIb/IIIa inhibitors | ✓ |  |  |
| Antiarrhythmics/rate control drugs | ✓ |  |  |
| NSAIDs | ✓ | ✓ | ✓ |
| Proton pump inhibitors | ✓ | ✓ | ✓ |
| P-gp inhibitors | ✓ | ✓ | ✓ |
| Hormone therapy | ✓ | ✓ | ✓ |
| Insulin | ✓ |  |  |
| **Intake of edoxaban** | ✓ | ✓ | ✓ |
| **Clinical events of interest after PCI** |  |  |  |
| Bleeding events^3^ | ✓ | ✓ | ✓ |
| Stroke^4^ | ✓ | ✓ | ✓ |
| MI | ✓ | ✓ | ✓ |
| PCI | ✓ | ✓ | ✓ |
| Death^5^ | ✓ | ✓ | ✓ |
| **HRU parameters** |  |  |  |
| Hospital admissions | ✓^6^ | ✓ | ✓ |
| Length of hospital stays | ✓ | ✓ | ✓ |
| **PRO (EQ-5D-5L)** | ✓ |  | ✓ |
| **ADRs** |  |  |  |
| Onset of event (relative to treatment with edoxaban) | ✓ | ✓ | ✓ |
| Event duration | ✓ | ✓ | ✓ |
| Severity and outcome | ✓ | ✓ | ✓ |

^1^Baseline or last available measurement in medical records (baseline or earlier). ^2^The following information was documented for each medication: daily dose, unit, start date, stop date or ongoing. For insulin, whether a patient did or did not take the medication was noted (yes, no or unknown).^3^Bleeding criteria used differed between baseline (ISTH bleeding criteria; **Table 4**) and follow-up (pre-defined bleeding event categories; **Table 5**) time points. ^4^Categorised as: any stroke; ischaemic stroke; haemorrhagic stroke; stroke (unknown type). ^5^Categorised as: all cause death; CV death; CV death sensitivity analysis i.e. unknown, other or missing reason for death will be considered as CV death). ^6^At baseline, hospital admissions from the preceding 12 months were recorded.

ACS, acute coronary syndrome; ADR, adverse drug reaction; AF, atrial fibrillation; COPD, chronic obstructive pulmonary disorder; CRNM, clinically relevant non-major; HF, heart failure; INR, International Normalised Ratio; ISTH, International Society on Thrombosis and Haemostasis; MI, myocardial infarction; NOAC, non-vitamin K antagonist oral anticoagulant; NSAID, non-steroidal anti-inflammatory drugs; NSTEMI, non-ST elevation myocardial infarction; NVAF, non-valvular atrial fibrillation; PCI, percutaneous coronary intervention; P-gp, P-glycoprotein; STEMI, ST elevation myocardial infarction; TIA, transient ischaemic attack; VHD, valvular heart disease; VTE, venous thromboembolic event.
